# Arbuscular mycorrhizal symbiosis alters the expression patterns of three key iron homeostasis genes, *ZmNAS1, ZmNAS3*, and *ZmYS1*, in S deprived maize plants

**DOI:** 10.3389/fpls.2015.00257

**Published:** 2015-04-20

**Authors:** Styliani N. Chorianopoulou, Yiorgos I. Saridis, Maria Dimou, Panagiotis Katinakis, Dimitris L. Bouranis

**Affiliations:** ^1^Plant Physiology and Morphology Laboratory, Crop Science Department, Agricultural University of AthensAthens, Greece; ^2^General and Agricultural Microbiology Laboratory, Crop Science Department, Agricultural University of AthensAthens, Greece

**Keywords:** maize, arbuscular mycorrhizal symbiosis, sulfur, iron homeostasis, nicotianamine synthase, yellow stripe

## Abstract

Nicotianamine is an essential molecule for Fe homeostasis in plants, its primary precursor is the S-containing compound methionine, and it is biosynthesized by the enzyme family of nicotianamine synthases (NASs). In maize, a graminaceous plant that follows Strategy II for Fe uptake, *ZmNAS* genes can be subgrouped into two classes, according to their roles and tissue specific expression profiles. In roots, the genes of class I provide NA for the production of deoxymugineic acid (DMA), which is secreted to the rhizosphere and chelates Fe(III). The Fe(III)-DMA complex is then inserted to the root via a ZmYS1 transporter. The genes of class II provide NA for local translocation and detoxification of Fe in the leaves. Due to the connection between S and Fe homeostasis, S deficiency causes Fe deprivation responses to graminaceous plants and when S is supplied, these responses are inverted. In this study, maize plants were grown in pots with sterile river sand containing FePO_4_ and were inoculated with the mycorrhizal fungus *Rhizophagus irregularis*. The plants were grown under S deficient conditions until day 60 from sowing and on that day sulfate was provided to the plants. In order to assess the impact of AM symbiosis on Fe homeostasis, the expression patterns of *ZmNAS1, ZmNAS3* (representatives of *ZmNAS* class I and class II), and *ZmYS1* were monitored before and after S supply by means of real time RT-PCR and they were used as indicators of the plant Fe status. In addition, total shoot Fe concentration was determined before and after S supply. AM symbiosis prevented Fe deprivation responses in the S deprived maize plants and iron was possibly provided directly to the mycorrhizal plants through the fungal network. Furthermore, sulfate possibly regulated the expression of all three genes revealing its potential role as signal molecule for Fe homeostasis.

## Introduction

Iron is an essential micronutrient for plants. Graminaceous plants follow the Strategy II for iron acquisition from the rhizosphere. Iron homeostasis in maize involves a series of processes, including the biosynthesis of deoxymugineic acid (DMA) for iron uptake from the rhizosphere and the translocation of iron throughout the plant body toward the sink organs (Kobayashi et al., 2006). In this iron uptake pathway, three molecules of S-adenosyl-methionine are combined to form nicotianamine (NA) which is then used as the precursor for DMA biosynthesis. DMA is secreted to the rhizosphere where it chelates Fe(III) and the complex DMA-Fe(III) is inserted into the root cell via the YS1 transporter (Curie et al., 2001; Nozoye et al., 2013). In addition to its role in iron uptake, NA plays also a dominant role in iron transfer, being used for the intercellular and intracellular Fe transport in all plants' organs, as well as long distance transport through the phloem (Kobayashi et al., 2006; Zhou et al., 2013b).

The primary precursor of NA is methionine, a sulfur-containing amino acid, so sulfur deprivation has a strong effect on iron homeostasis; as a result, S deficiency causes Fe deprivation responses to the graminaceous plants, which can be inverted when S is provided (Astolfi et al., 2003, 2010; Bouranis et al., 2003). The strong connection between sulfur and iron is typified by the Fe-S clusters, where most of the metabolically active Fe is bound to S. In chloroplasts, the most abundant Fe-S proteins are ferredoxin, photosystem I and cytochrome b_6_f complex. In mitochondria, major Fe-S proteins are complex I, II and III of the respiratory chain and aconitase in the citric acid cycle. This connection between the two nutrients suggests coordination between the metabolisms of S and Fe (Forieri et al., 2013; Vigani et al., 2013).

The enzyme family of nicotianamine synthases (NASs) produces NA using S-adenosyl-methionine as substrate molecule. Recent studies revealed the evolutionary relationship and tissue specific expression profiles of *ZmNAS* genes in maize leading to their grouping into two classes. The *ZmNASs* of class I are mainly expressed in the roots when iron is sufficient (Zhou et al., 2013a,b). *ZmNASs* of class I are mostly responsible for providing the precursor for DMA synthesis as well as for the long distance translocation of Fe in stem (Mizuno et al., 2003; Zhou et al., 2013b). The *ZmNASs* of class II are commonly accumulated in meristems and mesophyll cells of the leaves (Zhou et al., 2013a,b). These NAS genes are important for local iron distribution in leaves and sheaths and play a key role in iron homeostasis and detoxification (Mizuno et al., 2003; Zhou et al., 2013b). All *ZmNAS* genes of class I are induced as a response to Fe deficiency, while in Fe excess they are downregulated. On the other hand, all class II *ZmNASs* are downregulated in Fe deficiency and retain their expression levels or get overexpressed in Fe excess (Mizuno et al., 2003; Zhou et al., 2013b).

ZmYS1 is a membrane protein and functions as a proton-coupled symporter that mediates iron uptake in maize. ZmYS1 expression at both the mRNA and protein levels responds rapidly to changes in iron availability, whilst it is not regulated by zinc or copper deficiency (Curie et al., 2001; Roberts et al., 2004; Schaaf et al., 2004; Nozoye et al., 2013).

Arbuscular mycorrhizal (AM) symbiosis improves plant nutrient uptake under low nutrient availability (Bonfante and Genre, 2008). The role of AM symbiosis on phosphate has been extensively studied (Smith et al., 1994, 2003; Javot et al., 2007) and there seems to be a specific symbiotic phosphorus acquisition pathway (Harrison et al., 2002). Moreover, other nutrients such as nitrogen and sulfur are shown to be translocated from the fungal to the plant partner (Ames et al., 1983; Allen and Shachar-Hill, 2009; Leigh et al., 2009; Sieh et al., 2013). Few studies have been conducted in order to reveal the impact of AM symbiosis on iron uptake and the role of this symbiosis to plant iron homeostasis is still unclear. Studies on sorghum revealed that AM fungi can mobilize and/or take up Fe from soil and translocate it to the plant (Caris et al., 1998). However, other studies on maize propose that AM fungi increase total Fe in the shoot in the absence of other micronutrients and only in low P levels (Liu et al., 2000).

In this study, mycorrhizal and non-mycorrhizal plants were grown under prolonged sulfur deficiency in the presence of insoluble iron, as FePO_4_ salt. This salt has been used in previous studies, as practically insoluble form of phosphate, for the investigation of the impact of AM fungi on plant growth or P nutrition (Bolan et al., 1987; Virant-Klun and Gogala, 1995). In our study FePO_4_ had a dual role: it was the only source for Fe and the main source for P as the nutrient solution provided was P insufficient in order to promote the establishment of mycorrhizal symbiosis. Two months after sowing, sulfur was supplied to the plants, in the form of sulfate, to promote iron acquisition. The period before S supply was used for the determination of the impact of AM symbiosis on Fe status under long-term S deficient conditions while after S supply, components of the iron acquisition pathway were mainly investigated. The expression patterns of two *ZmNASs* were monitored in roots and leaves and *ZmYS1* was monitored in roots before and after sulfur repletion. Taking into account the expression profiles and tissue specific localization of *ZmNASs, ZmNAS1* was selected as representative of class I and *ZmNAS3* as representative of class II. In order to monitor the influence of AM symbiosis on Fe homeostasis, the expression patterns of *ZmNAS1* and *ZmYS1* in the roots, *ZmNAS3* in the leaves as well as total Fe concentrations in the shoots were used as indicators of the plants' iron status before and immediately after (24 and 48 h) sulfur supply.

## Materials and methods

### Plant material and growth conditions

Maize (*Zea mays* L., “Cisko,” Syngenta Hellas) seeds were thoroughly washed and placed on wet filter paper, in the dark at 28°C to germinate for 4 days. Then, the seedlings were transferred to batch culture boxes and grew hydroponically in well-aerated distilled H_2_O for the next 4 days. On day eight from sowing, all parts below the crown (i.e., mesocotyl and embryonic root system) as well as the seed were detached from the seedlings, which were thereafter, hydroponically grown in well-aerated nutrient solution, completely deprived of Fe and S and containing a low P concentration (10 μM), for 2 days. On day 10 from sowing, the seedlings were transferred to individual pots with sterile river sand (121°C for 1 h, 250 ml per pot) and the addition of practically insoluble FePO_4_(500 mg per pot). For the mycorrhizal treatment, 300 mg of *Rhizophagus irregularis* inoculum (synonym: *Glomus irregulare* DAOM197198, SYMPLANTA-001 standard grade, Symplanta) were added to each pot. The plants were watered two times a week with the Fe and S deficient nutrient solution until day 60 from sowing. Sulfur was provided in the form of sulfate on day 60, with the use of a nutrient solution only deprived of Fe. The nutrient solution deprived of Fe and S contained 5 mM KNO_3_, 10 μM KH_2_PO_4_, 2 mM Mg(NO_3_)_2_ 6H_2_O, 4 mM Ca(NO_3_)_2_ 4H_2_O, 0.86 mM CaCl_2_ 2H_2_O, 0.9 μM ZnCl_2_, 30 μM H_3_BO_3_, 0.9 μM CuCl_2_ 2H_2_O, 0.5 μM MoO3 85%, and 20 μM MnCl_2_ 4H_2_O. The iron-deficient nutrient solution applied on day 60 contained 5 mM KNO_3_, 10 μM KH_2_PO_4_, 2 mM Mg(NO_3_)_2_ 6H_2_O, 2.5 mM CaSO_4_ 2H_2_O, 1 mM MgSO_4_ 7H_2_O, 4 mM Ca(NO_3_)_2_ 4H_2_O, 0.9 μM ZnCl_2_, 30 μM H_3_BO_3_, 0.9 μM CuCl_2_ 2H_2_O, 0.5 μM MoO_3_ 85%, and 20 μM MnCl_2_ 4H_2_O. A controlled environment of 250 μmol photons m^−2^ s^−1^ photosynthetic photon flux density and a 14-h light photoperiod with day/night growth conditions at shoot base 28/23°C and RH 36/40% was used.

### Plant samplings for gene expression analysis

**Figure 1:**
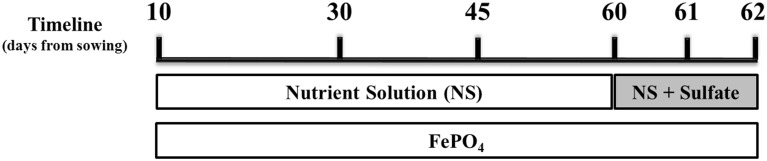
**Schematic illustration of the experimental design**. Mycorrhizal and non-mycorrhizal plants were grown under S deficient conditions until day 60; all plants were watered with a nutrient solution deprived of Fe and S and containing a low P concentration (10 μM). On day 60 S was provided to the plants as sulfate. Fe was provided to the plants in the form of sparingly soluble FePO_4_, throughout the experiment. Samplings took place on days 30, 45, 60 (before S supply), 61 and 62.

Samplings were performed on days 30, 45, 60, 61 (24 h after sulfur supply), and 62 (48 h after sulfur supply) from sowing and 3 h after the onset of light. The sampling of day 60 took place before the addition of sulfur. A schematic illustration of the experimental design, indicating also the days of the samplings, is presented in Figure 1. Lateral roots as well as two young expanding leaves were immediately frozen in liquid nitrogen and stored at −80°C until use. On each experiment, plant material from at least three biological replicates per treatment and sampling day was used. Lateral roots were chosen as root samples for the gene expression analysis because, according to Gutjahr and Paszkowski (2013), in roots of monocotyledon plants AM fungi preferentially colonize lateral roots. The young expanding leaves were chosen as strong sinks of Fe.

### Primer design

Performing Blast searches we identified the cDNA sequences of all recorded *ZmNAS* and *ZmYS* genes. Primers were designed for a representative of each class of the ZmNAS gene family as well as for *ZmYS1* (MaizeGDB: GRMZM2G156599). *ZmNAS1;1* (MaizeGDB: GRMZM2G385200) and *ZmNAS3* (MaizeGDB: GRMZM2G478568) were chosen for class I and II respectively. Real-time PCR primers were designed to amplify 100–200 bp fragments in the 3′untranslated regions, using the Primer Blast tool of NCBI. All primers were designed for 60°C annealing temperature and their sequences are as follows: *ZmNAS1;1*: Forward 5′-GGAACTTTTGAGCACCTATGCG-3′ and Reverse 5′-CACTTCACAATGCATAGCATCGAAT-3′; *ZmNAS3*: Forward 5′-CGTGTCTACACCACATGCGT-3′ and Reverse 5′-TCGGACTTCGACTTCTACCCT-3′; *ZmYS1*: Forward 5′-GTCTTCCATTCTCGCTCTGG-3′ and Reverse 5′-CAACCAACCACAGTTGATGC-3′. The gene of ubiquitin was used as an internal control and the target was detected by the following primers-pair: *ZmUBQ* (NCBI: NM_001138130): Forward 5′-TGTCTTCATGGCCAACCACT-3′ and Reverse 5′-GCTTGATAGGTAGGCGGGTG-3′.

### RNA extraction and real-time RT-PCR

Total nucleic acids were extracted from root and shoot samples using the Phenol-Chloroform protocol (Brusslan and Tobin, 1992) and were treated thereafter with Recombinant DNase I (RNase-free, Takara Bio Inc) in order to get the total RNA of each sample. An amount of 500 ng of RNA was reverse-transcribed using the PrimeScript RT reagent (Perfect Real Time, Takara Bio Inc).

Measurements of real-time RT-PCR were performed using KAPA SYBR FAST Master Mix (KAPA Biosystems) in the MxPro Mx3005P thermocycler (Stratagene, USA). The real-time RT-PCR was performed according to the respective protocol of the kit, using optical 96-well plates with the following PCR program: 95°C for 10 min, 40 cycles of 95°C for 30 s and 60°C for 1 min and the final cycle of 95°C for 1 min and 60°C for 30 s and 95°C for 30 s. Melting curve analysis was carried out after each amplification to exclude unspecific amplifications from the analysis. PCR amplification efficiencies were obtained using the LinRegPCR software (Ruijter et al., 2009). The relative expression ratios were calculated with the mathematical formula of Pfaffl (2001), using as reference the gene of ubiquitin and as targets the genes of *ZmNAS1, ZmNAS3*, and *ZmYS1*. As control the samples of day 30 (for the samplings before sulfur supply) or day 60 (for the samplings after sulfur supply) of the respective treatment were used.

### Verification of mycorrhizal colonization

In order to confirm the mycorrhizal colonization in the root samples two tests were performed, using both a histological and a molecular approach. Large and fine lateral roots were cleared in 2.5% KOH and stained with Trypan blue (Koske and Gemma, 1989) for fungal detection. For the molecular approach, using the total pre-DNase-treated nucleic acids extract, a PCR was performed with primers specific for the internal transcribed spacer 1 of the ribosomal RNA gene of *R. irregularis* (NCBI: JF820567). The sequences of the primers were the following ones: Forward 5′-TGATCTTTGATCATGGTTTCGC-3′ and Reverse 5′-TCGCACTTCGCTACGTTCTT-3′.

### Total Fe determination

Samples of maize shoots were used for the total Fe determination and were harvested on days 45, 60, and 65. The sampling of day 60 was conducted before sulfur supply. Samples were oven-dried at 80°C, the dry weight was recorded and the appropriate dry mass was ground to pass a 40 mesh screen using an analytical mill (IKA, model A10) prior to chemical analysis. Samples were digested with hot H_2_SO_4_ and repeated additions of 30% H_2_O_2_ until the digestion was complete, and thereafter, total Fe was determined in the diluted digests by atomic absorption spectrophotometry (GBC, Model Avanta spectrophotometer) (Mills and Jones, 1996).

### Statistical analysis

The experiment was performed two times under the same conditions and during two distinct time periods: autumn 2013 and spring 2014. Data were analyzed by *t*-test variance analysis with two-tailed distribution and two-sample unequal variance to determine the significance of differences among samplings.

## Results

### Verification of mycorrhizal colonization

**Figure 2:**
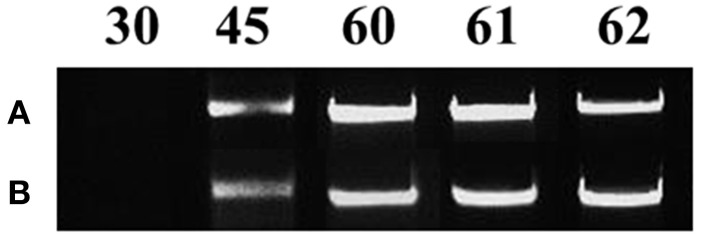
**Verification of mycorrhizal colonization via PCR amplification of the internal transcribed spacer 1 of the ribosomal RNA gene of *Rhizophagus irregularis* in the root samples of the mycorrhizal treatment**. Each amplification product illustrates a representative biological replicate of every sampling. Numbers indicate the respective sampling days. **A:** first repetition of the experiment (autumn 2013), **B:** second repetition of the experiment (spring 2014).

All mycorrhizal plants used were verified for the presence of *R. irregularis* prior to further analysis. Staining with Trypan blue showed that the roots of the maize plants were already colonized by the fungus on day 45. Arbuscules, vesicles and fungal hyphae were present in all large as well as fine lateral root samples examined. The molecular approach used to verify the mycorrhizal colonization revealed fungal presence in the root samples of mycorrhizal plants from day 45 until the end of the experiment (Figure 2). As depicted on Figure 2 the amplification products of the internal transcribed spacer 1 of the ribosomal RNA of *R. irregularis* have been comparable between the two repetitions of the experiment.

### Expression levels of *ZmNAS1* in the roots and *ZmNAS3* in the leaves

The expression profiles of *ZmNAS1* and *ZmNAS3* were monitored in both roots and leaves. The corresponding profiles of *ZmNAS1* in the leaves and *ZmNAS3* in the roots are provided as supplemental data (Figures S1, S2).

**Figure 3:**
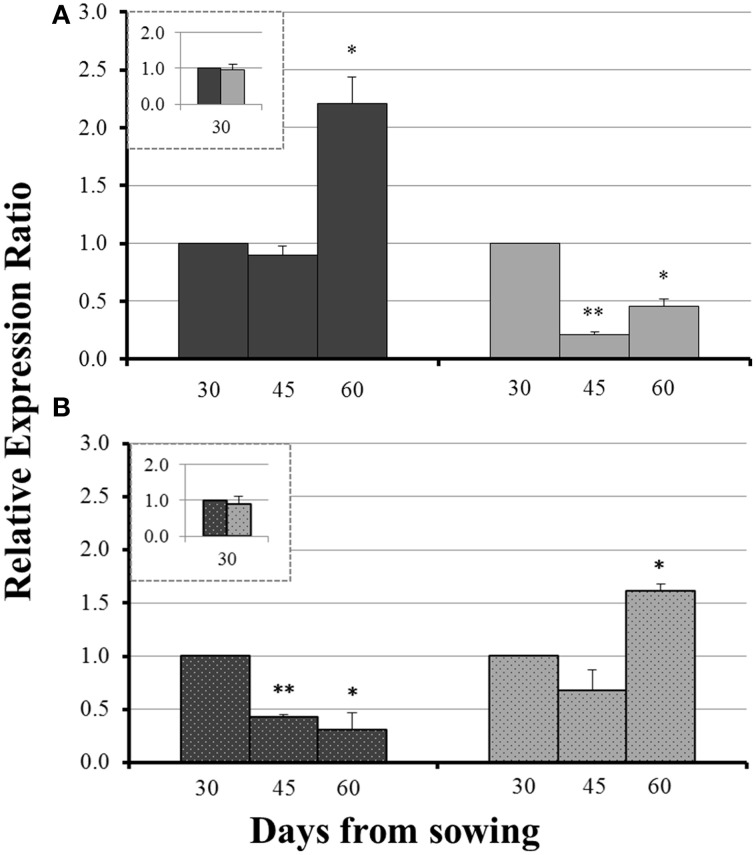
**Expression of *ZmNAS1* in the roots (A) and *ZmNAS3* in the leaves (B) of non-mycorrhizal (black columns) and mycorrhizal (gray columns) maize plants, before sulfur supply, relative to the expression of ubiquitin**. Day 30 of each treatment was used as control for the calculation of the relative expression ratios. The inset provides the relative expression ratio of each gene in the mycorrhizal plants on day 30, using the respective sample of non-mycorrhizal plants as control. Bars show the mean of the biological replicates ± SE, ^*^/^**^ indicated when the difference between the sampling and the respective control is statistically significant at *p* < 0.05/0.005 respectively.

On day 30 there was no significant difference in the expression levels of each gene between mycorrhizal and non-mycorrhizal plants (Figure 3, insets). Before sulfur supply, the roots of non-mycorrhizal plants revealed no significant change in the expression of *ZmNAS1* on day 45 which was followed by a significant upregulation on day 60 (Figure 3A). On the other hand, mycorrhizal roots showed a differential response; *ZmNAS1* was downregulated on both days 45 and 60 (Figure 3A). In the leaves of non-mycorrhizal plants, *ZmNAS3* was downregulated on both days before S repletion while the leaves of mycorrhizal plants showed an overexpression of *ZmNAS3* on day 60 (Figure 3B).

**Figure 4:**
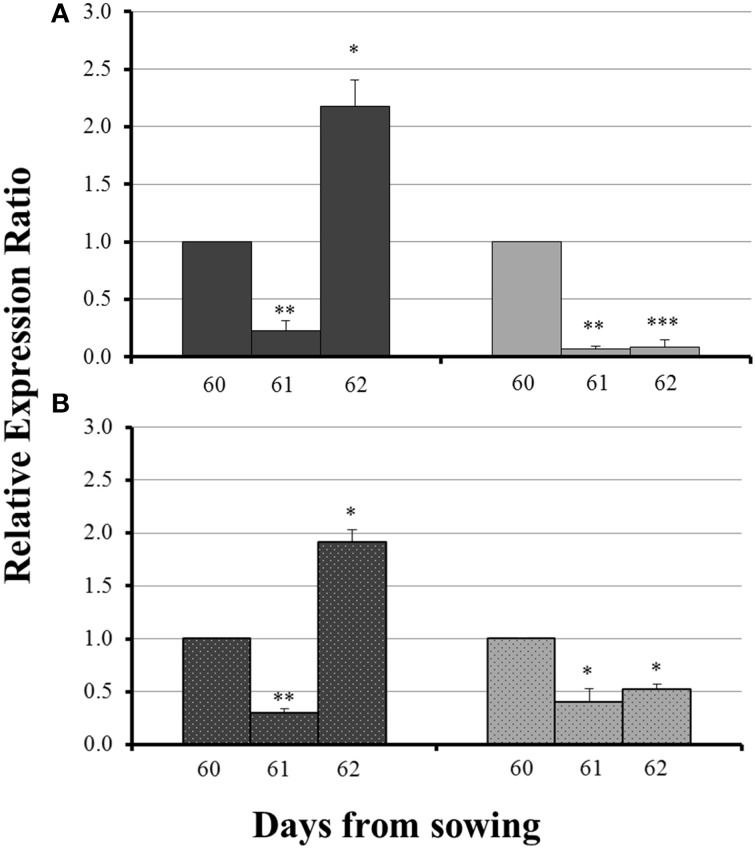
**Expression of *ZmNAS1* in the roots (A) and *ZmNAS3* in the leaves (B) of non-mycorrhizal (black columns) and mycorrhizal (gray columns) maize plants, after sulfur supply, relative to the expression of ubiquitin**. Day 60 of each treatment was used as control for the calculation of the relative expression ratios. Bars show the mean of the biological replicates ± SE, ^*/**^/^***^ indicated when the difference between the sampling and the respective control is statistically significant at *p* < 0.05/0.005/0.0005 respectively.

The influx of sulfate, 24 h after sulfur supply (day 61), resulted in a common response of both *ZmNAS1* in the roots and *ZmNAS3* in the leaves between mycorrhizal and non-mycorrhizal plants. Both genes were downregulated, 24 h after sulfur repletion, in all plants irrespective of the fungal presence (Figure 4). However, 48 h after sulfur supply (day 62), mycorrhizal plants showed again a differential response in relation to non-mycorrhizal plants. While in non-mycorrhizal plants both *ZmNAS1* in roots and *ZmNAS3* in leaves were upregulated, these genes presented a strong downregulation in the corresponding organs of mycorrhizal plants (Figure 4).

### Expression levels of *ZmYS1* in the roots

**Figure 5:**
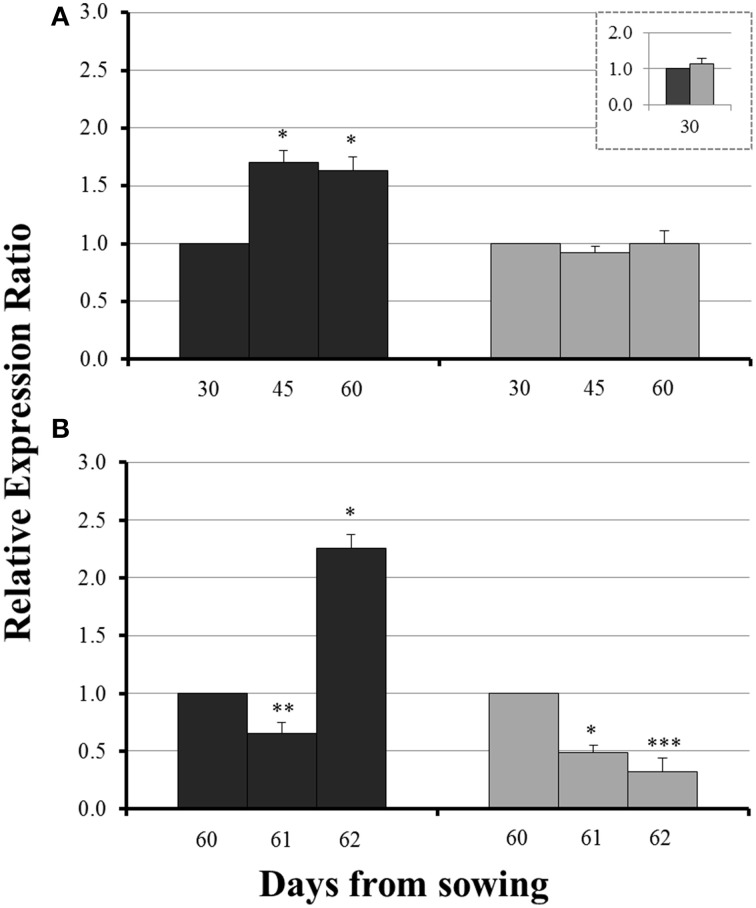
**Expression of *ZmYS1* in the roots of non-mycorrhizal (black columns) and mycorrhizal (gray columns) maize plants, before (A) and after (B) sulfur supply, relative to the expression of ubiquitin**. Day 30 or day 60 of each treatment was used accordingly as control for the calculation of the relative expression ratios. Bars show the mean of the biological replicates ± SE, ^*^/^**^/^***^ indicated when the difference between the sampling and the respective control is statistically significant at *p* < 0.05/0.005/0.0005 respectively.

No significant difference in the expression levels of *ZmYS1* between mycorrhizal and non-mycorrhizal roots was monitored on day 30 (Figure 5, inset). Before sulfur supply, *ZmYS1* was significantly overexpressed in the roots of non-mycorrhizal plants (Figure 5A). On the other hand, mycorrhizal roots did not show any considerable response and the expression ratios of *ZmYS1* remained stable until day 60 (Figure 5A). The influx of sulfate, 24 h after sulfur supply (day 61), resulted again in a downregulation of *ZmYS1* in the roots of all plants (Figure 5B). However, 48 h after sulfur supply (day 62), mycorrhizal plants revealed once more a differential response against non-mycorrhizal plants. *ZmYS1* was strongly upregulated in non-mycorrhizal roots while mycorrhizal roots presented an intense downregulation (Figure 5B).

### Total Fe concentrations in the shoots

Total Fe concentration of the aerial part of non-mycorrhizal plants decreased significantly from day 45 to day 60. In the mycorrhizal plants, the corresponding concentrations of total Fe presented no significant change, although the dry mass was increasing (data not shown). On day 65, i.e., 5 days after the addition of sulfate, total Fe concentration increased in the shoots of all plants. Interestingly, Fe concentration in mycorrhizal plants on that day was found to be 3.4 times higher than the corresponding one of non-mycorrhizal plants (Table 1).

**Table 1:**
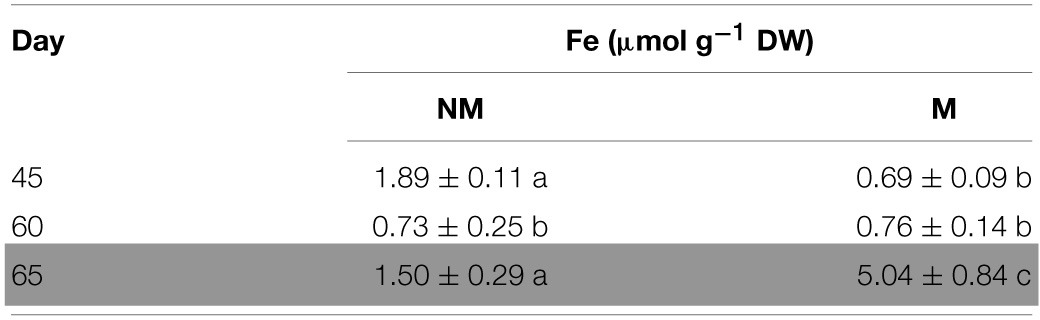
**Time course of total Fe concentrations (mean values ± SE) in the shoots of mycorrhizal (M) and non-mycorrhizal (NM) maize plants**.

*Different letters represent statistically significant differences (P < 0.05). The grey row indicates the period after sulfur supply*.

## Discussion

### Mycorrhizal colonization prevents Fe deprivation responses in S deprived maize plants

AM symbiosis modifies the nutrient status of the plants; however the impact of AM colonization on the plants' iron status has not been clarified, yet. Only a few studies on graminaceous plants have shown a potential contribution of AM fungi in Fe uptake and they aimed to examine whether and/or how much Fe is transferred to the mycorrhizal plants in contrast to non-mycorrhizal plants (Caris et al., 1998; Liu et al., 2000). In our work, three key Fe homeostasis genes were used for the assessment of the effect of AM symbiosis on Fe homeostasis in maize plants.

Class I *ZmNAS* genes are mainly expressed in the roots and play a crucial role in the Strategy II Fe acquisition pathway, providing the essential NA molecules for the formation of DMA. The genes of class II are mostly expressed in the leaves and are involved in the short distance translocation and/or detoxification of Fe (Mizuno et al., 2003; Zhou et al., 2013a,b). When Fe is depleted, class I genes are induced, promoting the Strategy II Fe acquisition pathway and class II genes are downregulated, possibly because under such conditions there are lower needs of Fe transport in the leaves. Moreover, the gene of the Fe(III)-DMA chelate transporter, *ZmYS1*, is strongly and rapidly induced in Fe deprived conditions (Roberts et al., 2004). However, when Fe is in excess, class I *ZmNAS* genes are downregulated and the genes of class II either retain their expression levels or get overexpressed (Zhou et al., 2013b). *ZmNAS1* and *ZmNAS3* are members of class I and class II, respectively, so their expression patterns follow the expression profiles mentioned above, when Fe is either depleted or in excess (Table 2).

**Table 2:** Alterations in the expression profiles of *ZmNAS1, ZmNAS3*, and *ZmYS1* as response to Fe status (according to the literature^*^) and the treatments conducted in this study before S supply.

	**Fe status**	***ZmNAS1* roots**	***ZmNAS3* leaves**	***ZmYS1* roots**	
Literature	Deprivation	↑	↓	↑	
	Sufficiency/excess	↓	~ ↑	~	
		***ZmNAS1* roots**	***ZmNAS3* leaves**	***ZmYS1* roots**	**Fe concentration**
Treatments	NM	↑	↓	↑	▾
	M	↓	↑	~	■

*NM: non-mycorrhizal plants, M: mycorrhizal plants, ↑: upregulation of the gene, ↓: downregulation of the gene, ~: no significant change in the expression, ■: decrease in shoot Fe concentration, ▾: no significant change in shoot Fe concentration*.

^*^The data provided for the expression profiles of the three genes as response to the Fe status were taken from Mizuno et al. (2003); Roberts et al. (2004), and Zhou et al. (2013b).

In this study, sulfur deficient conditions had a strong, negative impact on the Strategy II Fe acquisition pathway. As proposed by previous studies, S deprivation generates Fe deficiency in graminaceous plants (Astolfi et al., 2003; Bouranis et al., 2003). Before S supply, non-mycorrhizal plants showed an anticipated response to S depletion; the concentration of total Fe in the shoots reduced from day 45 to day 60 (Table 2), probably due to a dilution effect. In addition, *ZmNAS1* was induced on day 60 in the roots and *ZmNAS3* was downregulated in the leaves (Table 2). The expression profiles of these genes in the non-mycorrhizal plants suggest that these plants sensed Fe deprivation and the reducing Fe concentration confirms that the plants had experienced a difficulty in taking up Fe from the provided sparingly soluble FePO_4_ salt. On the other hand, mycorrhizal plants revealed a completely diverse response in the sulfur deficient conditions. *ZmNAS1* was downregulated in the roots and *ZmNAS3* was upregulated in the leaves on day 60, which in turn suggests that mycorrhizal plants probably sensed Fe sufficient or excessive conditions (Table 2). Moreover, total shoot Fe concentrations remained at the same levels from day 45 to day 60 (Table 2), suggesting that mycorrhizal plants took up Fe. Especially on day 60, although shoot Fe concentrations were equal between mycorrhizal and non-mycorrhizal plants, the expression profiles of the two genes were exactly the opposite between the treatments, showing that the same iron concentration is sensed as sufficient for the mycorrhizal and insufficient for the non-mycorrhizal plants.

The above divergent observations can be supported by the expression profiles of *ZmYS1*. The expression levels of this gene respond rapidly to the changes of the plant Fe status (Roberts et al., 2004). An upregulation of *ZmYS1*, observed under Fe deprived conditions, was also observed in the roots of non-mycorrhizal plants before S supply, clearly suggesting that these plants sensed Fe insufficiency (Table 2). In contrast, mycorrhizal roots sustained the expression levels of *ZmYS1* until day 60 a fact that endorses the expression profiles of *ZmNAS1* and *ZmNAS3*. The expected Fe deprivation responses in the S deprived conditions were prevented because of the AM symbiosis, whilst S deprivation obstructed Fe absorption and/or translocation in non-mycorrhizal plants leading them to sense Fe deficient conditions. Consequently, it is suggested that the combined expression patterns of *ZmNAS1* and *ZmYS1* in the roots and *ZmNAS3* in the leaves can be used as indicators of the plant Fe status (Table 2).

### Mycorrhizal symbiosis alters the Fe uptake pathway in maize plants

AM symbiosis severely alters the processes of nutrient uptake according to the needs of the mycorrhizal plants. Phosphorus, for instance, is provided to the mycorrhizal plants through the fungus and there was recorded loss of function of the direct P uptake pathway in the colonized roots (Smith et al., 2003). In this study, *ZmNAS1* and *ZmYS1*, key genes of the Strategy II Fe uptake pathway, were used in order to determine the impact of AM symbiosis on the plant Fe acquisition pathway.

Forty-eight hours after sulfur supply (day 62), mycorrhizal and non-mycorrhizal plants showed again completely diverse responses. The overexpression of *ZmNAS1* in the roots of non-mycorrhizal plants, 48 h after S supply (Figure 4A), was anticipated as S supply contributes in the production of DMA by promoting the Strategy II Fe acquisition pathway (Astolfi et al., 2010). The severe overexpression of *ZmYS1* in the roots (day 62) as well as the increase of total Fe concentration in the shoots of non-mycorrhizal plants (day 65) sustain this hypothesis (Figure 5B, Table 1). On the other hand, mycorrhizal plants did not show an intention to enhance the Strategy II pathway, even if S was provided. The downregulation of both *ZmNAS1* and *ZmYS1* in the roots (Figures 4A, 5B) as well as the increased shoot Fe levels on day 65 (Table 1), suggest that Fe was mainly transported directly to them by the AM fungus in a special, symbiotic Fe uptake pathway. Further analyses should be conducted in order to confirm the existence of such a pathway.

### Sulfate probably regulates the expression of *ZmNAS1, ZmNAS3*, and *ZmYS1*

Sulfur and iron metabolisms are highly interrelated and there is a co-regulation between their uptake pathways (Forieri et al., 2013; Vigani et al., 2013). As depicted in Figures 4, 5, 24 h after sulfate supply (day 61), there was a common response in the expression of ZmNAS1, ZmNAS3, and *ZmYS1* of all plants. All genes were suppressed and such suppression, 24 h after S supply, is difficult to be explained. We assume that the incoming sulfate probably played a role as signal molecule for Fe homeostasis throughout the plant body, a role which has been previously given to sulfate by Forieri et al. (2013). The exact way by which the expressions of these genes are regulated by sulfate, as well as the potential impact of this signaling procedure remains unknown and if this is the case, it is of great interest for future analyses.

## Concluding remarks

AM symbiosis managed to prevent Fe deprivation responses in S deprived maize plants. It is suggested that Fe was provided directly to the mycorrhizal plants through the fungal network. Moreover, sulfate probably regulates the expression of three key Fe homeostasis genes in maize providing a hint of its role as a signal molecule.

### Conflict of interest statement

The authors declare that the research was conducted in the absence of any commercial or financial relationships that could be construed as a potential conflict of interest.

## Abbreviations

AM: arbuscular mycorrhiza
AMF: arbuscular mycorrhizal fungi
DMA: deoxymugineic acid
NA: nicotianamine
NAS: nicotianamine synthase
YS: yellow stripe.
