# Supplementary material for: Arbuscular mycorrhizal symbiosis alters the expression patterns of three key iron homeostasis genes, ZmNAS1, ZmNAS3, and ZmYS1, in S deprived maize plants
Source: Front Plant Sci. 2015 Apr 20;6:257. doi: 10.3389/fpls.2015.00257 (PMC4403604; doi:10.3389/fpls.2015.00257)
Supplement: Supplementary file 1 [file DataSheet1.DOCX]

**Supplementary material of the paper**

**Arbuscular mycorrhizal symbiosis alters the expression patterns of three key iron homeostasis genes, ZmNAS1, ZmNAS3 and ZmYS1, in S deprived maize plants**

**Styliani N. Chorianopoulou^1^*, Yiorgos I. Saridis^1^, Maria Dimou^2^, Panagiotis Katinakis^2^ and Dimitris L. Bouranis^1^**

^1^Plant Physiology and Morphology Laboratory, Crop Science Department, Agricultural University of Athens, Greece

^2^General and Agricultural Microbiology Laboratory, Crop Science Department, Agricultural University of Athens, Greece

*** Correspondence:** Styliani N. Chorianopoulou, Plant Physiology and Morphology Laboratory, Crop Science Department, Agricultural University of Athens, Iera Odos 75, 11855 Athens, Greece, [s.chorianopoulou@aua.gr](mailto:s.chorianopoulou@aua.gr)

**Figure Legends**

**Figure S1. Expression of *ZmNAS1* in the leaves (A) and *ZmNAS3* in the roots (B) of non-mycorrhizal (black columns) and mycorrhizal (grey columns) maize plants, before sulfur supply, relative to the expression of ubiquitin.** Day 30 of each treatment was used as control for the calculation of the relative expression ratios. The inset provides the relative expression ratio of each gene in the mycorrhizal plants at day 30, using the respective sample of non-mycorrhizal plants as control. Bars show the mean of the biological replicates ± SE, */** indicated when the difference between the sampling and the respective control is statistically significant at p<0.05/0.005 respectively.

**Figure S2. Expression of *ZmNAS1* in the leaves (A) and *ZmNAS3* in the roots (B) of non-mycorrhizal (black columns) and mycorrhizal (grey columns) maize plants, after sulfur supply, relative to the expression of ubiquitin.** Day 60 of each treatment was used as control for the calculation of the relative expression ratios. Bars show the mean of the biological replicates ± SE, */** indicated when the difference between the sampling and the respective control is statistically significant at p<0.05/0.005 respectively.
