# Supplementary figures and images for: Arbuscular mycorrhizal symbiosis alters the expression patterns of three key iron homeostasis genes, ZmNAS1, ZmNAS3, and ZmYS1, in S deprived maize plants
Source: Front Plant Sci. 2015 Apr 20;6:257. doi: 10.3389/fpls.2015.00257 (PMC4403604; doi:10.3389/fpls.2015.00257)

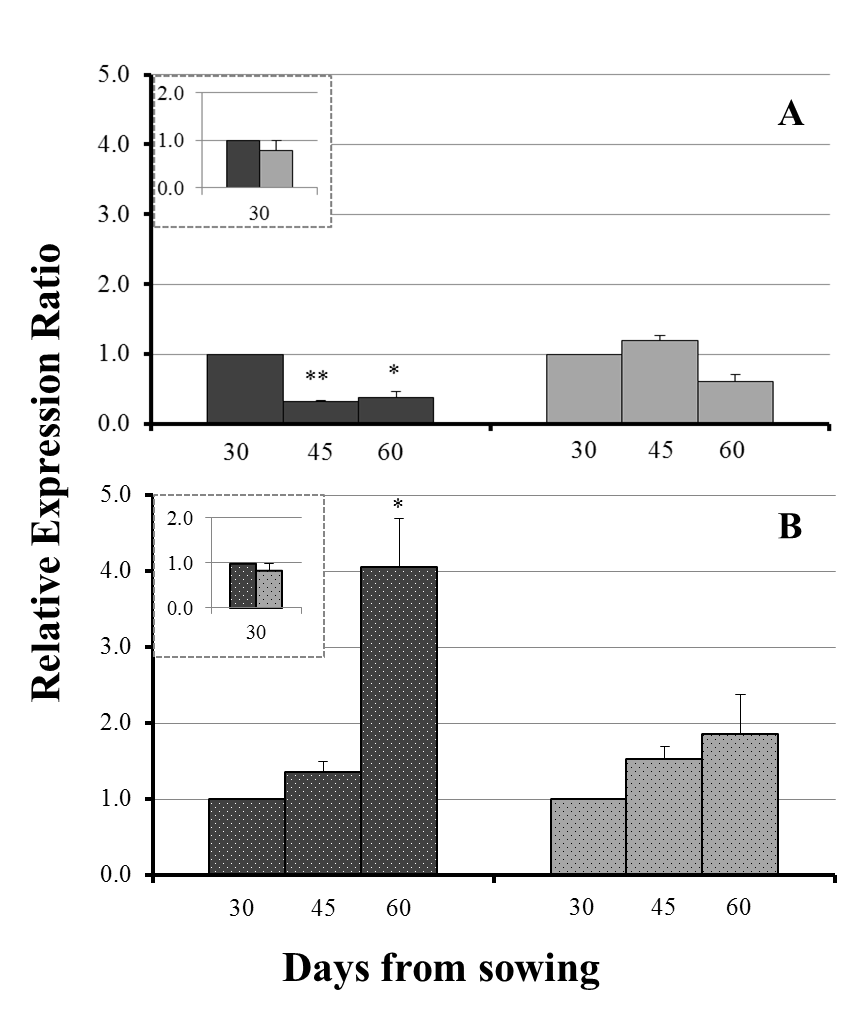

Supplement: Supplementary file 2 [file Image1.TIF]

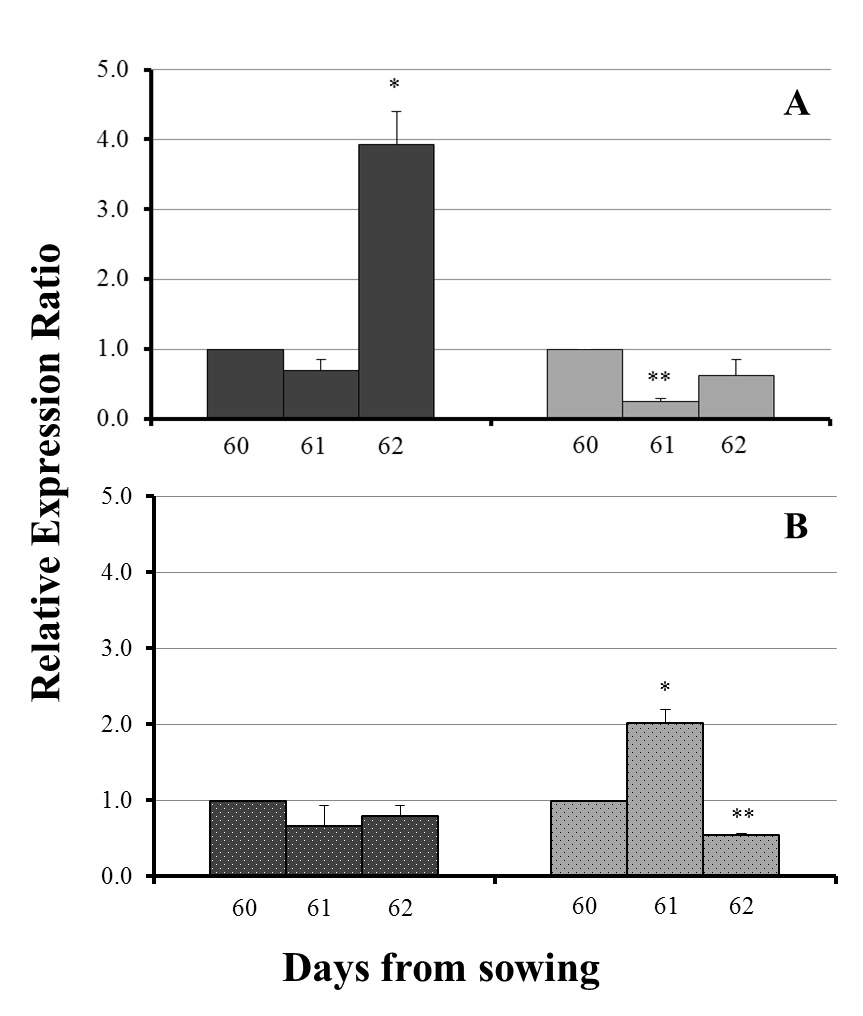

Supplement: Supplementary file 3 [file Image2.TIF]
